# First Brazilian Series of Urologic Surgeries Using Versius Robotic Multiport Platform

**DOI:** 10.1590/S1677-5538.IBJU.2025.0130

**Published:** 2025-07-25

**Authors:** Gilberto Laurino Almeida, Wilson F. Schreiner Busato, Andre Kives Berger, Vicente Codagnone, Gustavo Winter, Roberto Kinchescki, Phelipe Celestino Santos, Leticia Mendes Leães, Yoann Pierre Pérès, Daniel Gobbi, Artur de Oliveira Paludo, Lucas Medeiros Burttet, Felipe Guimarães Pugliesi, Gustavo Cardoso Guimarães, Gustavo Schroeder, Aguinel Jose Bastian, Luis Felipe Piovesan, Milton Berger, Ricardo Kupka da Silva, Fabrício G. Kaminagakura

**Affiliations:** 1 Hospital Unimed Litoral Balneário Camboriú SC Brasil Hospital Unimed Litoral, Balneário Camboriú, SC, Brasil; 2 Hospital Moinhos de Vento Porto Alegre RS Brasil Hospital Moinhos de Vento, Porto Alegre, RS, Brasil; 3 Imperial Hospital de Caridade Florianópolis SC Brasil Imperial Hospital de Caridade, Florianópolis, SC, Brasil; 4 Hospital São Vicente de Paulo Passo Fundo RS Brasil Hospital São Vicente de Paulo, Passo Fundo, RS, Brasil; 5 Hospital São Luiz Itaim São Paulo SP Brasil Hospital São Luiz Itaim, São Paulo, SP, Brasil; 6 Hospital Beneficência Portuguesa de São Paulo São Paulo SP Brasil Hospital Beneficência Portuguesa de São Paulo, São Paulo, SP, Brasil

**Keywords:** Robotic Surgical Procedures, Minimally Invasive Surgical Procedures, Urology

## Abstract

**Purpose:**

This is the first series of urological conditions using the Versius Surgical System® platform in Brazil. We evaluated perioperative results, and surgeons’ learning curve in Brazilian hospitals.

**Materials and Methods:**

This prospective, multicenter study included urological surgeons certified on the Versius® platform and their respective procedures from May 2022 to August 2024. Perioperative variables included type of surgery, docking time, console time, number of bedside units (BSUs), complications according to the Clavien–Dindo classification, readmission and reoperation rates within 30 days after surgery. Surgeons were stratified according to experience in laparoscopy and robotic surgery.

**Results:**

Nineteen surgeons and 302 urological procedures were included. There was no statistically significant difference between docking time and console time when comparing obese patients (BMI > 30, p=0.507) and non-obese patients (BMI < 30, p=0.733). One hundred thirty-seven (45.4%) surgeries were performed by surgeons with experience in laparoscopy and robotics (> 5 years), and 238 surgeries (78.8%) were performed by surgeons who had more than 10 Versius® procedures. Docking time was 13.4 (SD 7.23) minutes. Nephroureterectomy was the procedure with the shortest docking (8.7 minutes, 6–13 min) and console (83.3 minutes, 38–152 min) times. Of the 10 surgeons with 10 or more procedures, 6 had shorter docking times, and 4 had shorter console times. There were four Clavien–Dindo III-IV complications within 30 days postoperatively (1.3%), and six (2%) patients required reoperation. Mean hospital stay was 2 days.

**Conclusion:**

This study is the largest Brazilian and worldwide case series of urological surgeries performed using the Versius® robotic platform. Our results demonstrate the safety and efficacy of the Versius® platform and provide real-world evidence of the surgeons’ learning curves and performance based on their prior expertise in laparoscopic and/or robotic surgery.

## INTRODUCTION

The field of minimally invasive surgery, particularly in Urology, evolved significantly during the early 2000s when the Da Vinci® robotic platform (Intuitive Surgical, Sunnyvale, CA, USA) was approved by the Food and Drug Administration (FDA) in the United States. Despite the benefits demonstrated by the robotic technique for patients, over the last 20 years, the Da Vinci® platform has been used in a still limited number of patients globally (1, 2). However, several new multiport and single-port robotic platforms have been developed, and some are now clinically available (2).

The Versius Surgical System® (CMR, Cambridge, United Kingdom) has innovative features that differ from the original Da Vinci® concept. It is a new teleoperated surgical robotic system intended for use of robotic assisted surgery with an ergonomic open console and modular configuration of arms. Moreover, the open design of the console has the advantage of facilitating communication between the team, and the manual controls provide all functions, including cutting and coagulation energies, without pedals attached to the console (3, 4). The Versius® arm is designed to mimic the human arm and wrist. Instruments reach up to seven degrees of freedom, which results in a better dissection and surgical approach than laparoscopy. The operating room does not require specialized infrastructure to accommodate the system, and the instruments are sterilized in a standard autoclave (5).

In Brazil, Versius® was registered for clinical use with ANVISA (National Health Surveillance Agency) in July 2021 (Registration 81346500064), and the first surgery was performed on April 19, 2022. Currently, there are 10 Versius® platforms installed in eight Brazilian states [Rio Grande do Sul (2), Santa Catarina (2), São Paulo (1), Rio de Janeiro (1), Bahia (1), Espirito Santo (1), Mato Grosso (1) and Federal District/Brasília (1)]. More than 1500 patients underwent Versius® surgical procedures by different surgical specialties, including Urology (cmrsurgical.com). Our hypothesis is Verisus® a safe platform and the learning curve is feasible for Brazilian surgeons? This is the first Brazilian series of urological surgeries using the Versius® platform and evaluates this platform's perioperative results, learning curve and safety in the Brazilian reality.

## MATERIALS AND METHODS

This prospective, multicenter study describes the clinical experience of Brazilian urological surgeons qualified and certified on the Versius® system, according to CMR surgical's training. These surgeons were located at six private institutions located in the South and Southeast regions of Brazil (São Paulo, Santa Catarina and Rio Grande do Sul). Urological surgeons were invited to participate in the study as soon as they were qualified and certified on this robotic platform. If accepted, they signed the Free and Informed Consent Form and received guidance on completing the data collection instrument. Data collection took place between May 2022 and August 2024. This study was approved by the Research Ethics Committee of Instituto Presbiteriano Mackenzie, CAAE no. 80018524.0.0000.0103.

**Figure 1:**
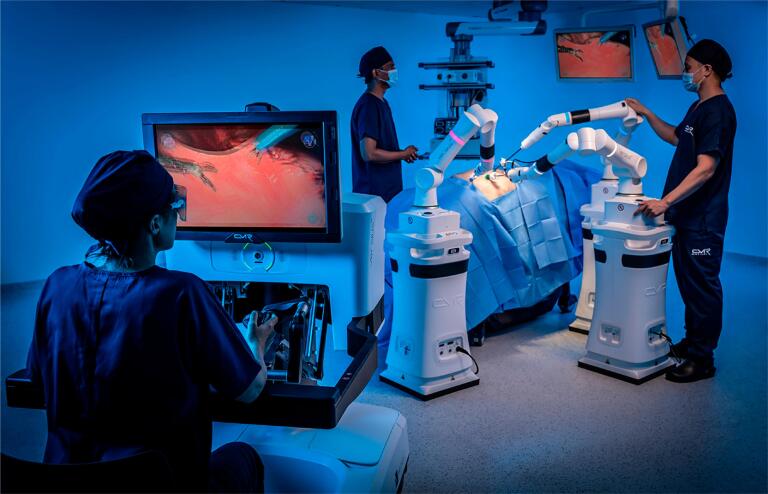
Console and bedside units.

This Versius® system comprises the surgeon's console with hand controllers to manipulate the arms and camera of the four robotic arms [bedside units (BSUs)] (Figure-1).

**Figure 2:**
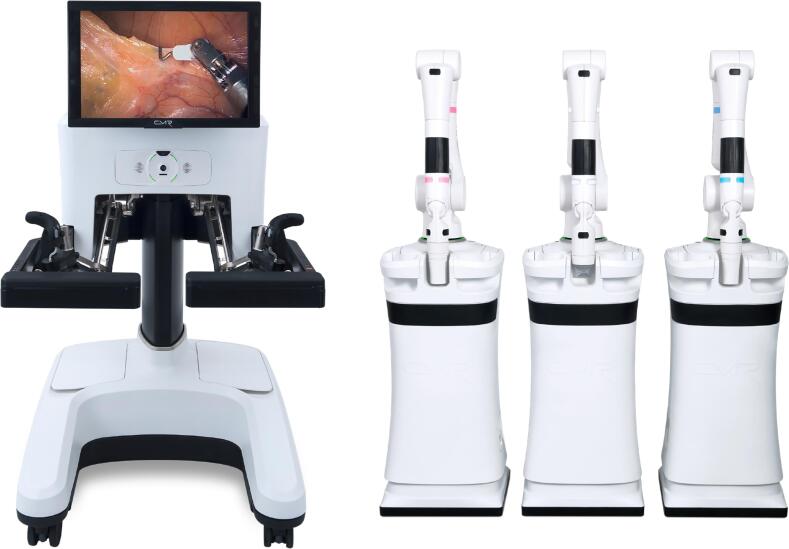
Open Console with no pedals and docking of modular arms (BSUs).

The modular configuration is independent of the BSUs, providing several docking options depending on the patient's characteristics and the surgical team's needs as well as a high flexibility in port-placement following the surgeon to replicate as far as possible the setup of conventional laparoscopic surgery. The surgeon receives three-dimensional high-definition video-feedback from the camera via head-up display (Figure-2). As a disruptive system due to the difference of Da Vinci system, the docking with modular arms and docking with an open console without pedals (Figure-2) could impact in the learning curve.

A prospective, non-consecutive database was developed, including pre-, intra-, and postoperative variables completed by the surgeons responsible for each surgical case, which were obtained through the CMR Surgical data manager system. The variables of interest included type of surgery, docking time, console time, number of BSUs used, complications according to the Clavien–Dindo classification, readmission within 30 days and reoperation within 30 days.

Surgeons were stratified according to their experience in laparoscopy and robotic surgery (laparoscopy < and > 5 years, robotics < 5 years, laparoscopy and robotics < and > 5 years) to assess the impact of prior experience on the learning curve.

## STATISTICAL METHODS

The data were cataloged in Excel and later exported to IBM SPSS statistics version 20.0 for statistical analysis. The normality of the variables was verified using the Kolmogorov–Smirnov test. The variables with a normal distribution were described using the mean and standard deviation, and those with asymmetric distribution were described by the median and interquartile range. The qualitative variables were described by frequencies and percentages.

## RESULTS

Of the 24 qualified and certified surgeons at these locations, 19 (79.1%) agreed to participate in the study, reporting 302 urological procedures from May 2022 to August 2024. The hospital A included 4 (20%) surgeons and 143 (47,4%) procedures, B included 7 (35%) surgeons and 109 (36.1%) procedures, C included 5 (25%) surgeons and 32 (10.6%) procedures, D included 1 (10%) surgeon and 10 (3.3%) procedures, E included 2 (5%) surgeons and 6 (2%) procedures, F included 1 (5%) surgeon and 2 (0.7%) procedures. One surgeon performed surgery at institutions E and F.

Table-1 shows the distribution of surgeons according to their previous experience in laparoscopy and/or robotics, the number of surgeries per surgeon, the different robotic procedures performed in this case series and the distribution of procedures performed according to docking time and console time.

**Table 1:** Distribution of surgeons according to previous experience, number of procedures per surgeon, types of procedure performed, and procedures performed according to docking time and console time.

Variables			
**Surgeons Experience**		N	%
	Laparoscopy and robotics > 5 years	137	45.4
	Laparoscopy and robotics < 5 years	55	18.2
	Robotics < 5 years	57	18.9
	Laparoscopy > 5 years	43	14.2
	Laparoscopy < 5 years	10	3.3
**Number of procedures per surgeon**		**N**	**%**
	2–5	8	42.1
	6–10	4	21.1
	11–20	4	21.1
	> 21	3	15.7
**Types of Procedures**		**N**	**%**
	RARP*	183	60.6
	Partial nephrectomy	53	17.5
	RASP^#^	11	3.6
	Radical nephrectomy	9	3.0
	Adrenalectomy	8	2.6
	Colpopromontofixation	8	2.6
	Nephroureterectomy	7	2.3
	Pyeloplasty	7	2.3
	Radical cystectomy	3	1.0
	Retroperitoneal lymphadenectomy	3	1.0
	Others	10	3.3
**Procedure**		**Docking Time Average/SD [Min–Max]**	**Console Time Average/SD [Min–Max]**
	RARP	12.99 ±7.3 [4**–**45]	196.7 ±74.2 [49**–**540]
	RASP	13.8 ±5.4 [7**–**23]	160.2 ±54.3 [104**–**243]
	Partial nephrectomy	13.0 ±5.9 [4**–**30]	110.5 ±46.8 [38**–**234]
	Total nephrectomy	15.0 ±4.7 [9**–**21]	126.0 ±43.8 [80**–**223]
	Nephroureterectomy	8.7 ±2.8 [6**–**13]	83.3 ±40.4 [38**–**152]
	Pyeloplasty	13.3 ±5.4 [6**–**20]	116.4 ±42.7 [65**–**189]
	Adrenalectomy	15.6 ±8.7 [7**–**35]	113.1 ±42.9 [50**–**175]
	Colpopromontofixation	22.6 ±15.4 [12**–**60]	151.2 ±49.2 [90**–**236]
	Radical cystectomy	14.0 ±4.0 [10**–**18]	238.7 ±3.5 [235**–**242]
	Lymphadenectomy	14.0 ±7.0 [9**–**22]	128.3 ±119.9 [30**–**262]
	Others	13.3 ±5.5 [5**–**23]	121.4 ±114.8 [23**–**420]

The study sample included 259 (85.8%) male and 43 (14.2%) female patients, with a mean age of 63.8 years (SD 12.6; 21–86) and mean BMI of 27.3 (SD 4.6; 16.6–43.4). There was no statistically significant difference between docking time and console time when comparing surgeries on obese (BMI > 30, p=0.507) and non-obese (BMI < 30, p=0.733) patients.

Regarding the number of BSUs utilized, four were used in 270 (89.4%), and three were used in 32 (10.6%) cases. The most common BSU arrangement was three on the left and one on the patient's right side. This configuration was used in 78 pelvic procedures (25.85%). It was also found that in 240 (79.5%) cases, five access points (four for the robotic forceps and one for the auxiliary laparoscopic) were utilized.

As shown in Table-1, the mean time for BSU docking was 13.4 (SD 7.23) minutes. Moreover, the nephroureterectomy procedure had the shortest docking (mean 8.7 minutes, 6-13 min) and console (mean 83.3 minutes, 38–152 min) times.

Since 10 (52.6%) surgeons had performed ≥10 procedures, we performed statistical analyses to verify whether there was a correlation between the reduction in docking time and console time as the surgeon progressed along the robotic platform learning curve (Table-2). The surgeon's previous experience was not considered in the correlation test, but it is described in the table to explain these results. The values presented after the p-value refer to the magnitude, which, when negative, indicates that as surgeons gain experience with each procedure, the time reduces; greater than 0.6 is strong, and 0.4–0.6 is considered moderate. Among the 10 surgeons with ≥10 procedures, docking time decreased for six and console time decreased for four as the learning curve developed.

**Table 2:** Distribution of the correlation between the reduction in docking time and console time according to the surgeon's experience for each procedure.

Surgeon	Experience	N surgeries	Docking Time		Console Time	
			**r**_s_	*p*	**r**_s_	*p*
11	LAP < 5 years	10	-0.162	0.678	-0.400	0.286
4	LAP > 5 years	10	-0.163	0.653	-0.167	0.668
7	LAP > 5 years	17	-0.648	**0.007**	-0.557	**0.020**
17	LAP > 5 years	15	-0.869	**0.0001**	0.084	0.766
18	ROB < 5 years	51	-0.700	**0.0001**	-0.90	0.540
2	LAP and ROB < 5 years	14	-0.691	**0.006**	-0.130	0.659
10	LAP and ROB < 5 years	23	0.341	0.112	-0.541	**0.008**
15	LAP and ROB < 5 years	12	0.836	**0.001**	-0.719	**0.008**
19	LAP and ROB < 5 years	10	-0.948	**0.0001**	-0.236	0.511
6	LAP and ROB > 5 years	106	-0.292	**0.002**	-0.207	0.034

LAP = laparoscopy; ROB = robotic surgery.

Only surgeons with > 10 surgeries were included in the analysis

* Spearman correlation coefficient between variables

Concerning postoperative complications within 30 days, according to the Clavien–Dindo classification, there were 285 (94.4%) classified as I, 12 (4.0%) as II, 1 (0.3%) classified as III, and 3 (1.0%) classified as IV. Of the 302 patients, only 8 (2.6%) were readmitted to the hospital in less than 30 days postoperatively, and 6 (2%) required reoperation within 30 days. The mean hospital stay was 2 (median 2.23) days. There was one conversion to open surgery due to intraoperative bleeding caused by the release of a hemostatic clip near the aorta.

## DISCUSSION

The Versius® platform had its first clinical case performed in India in March 2019, and currently, there are approximately 170 platforms installed in more than 30 countries, with 29,000 surgeries performed worldwide. In Brazil, there are approximately 1500 surgeries performed in different specialties (cmrsurgical.com); however, since the beginning of its clinical use, no urological case series have been reported to date, making this the largest case series of urological surgeries described with the Versius® platform reported in the literature. Soumpasis et al. (6) recently published a cohort of 2083 patients operated on with Versius® in an international multicenter study covering several surgical specialties; however, there were only 169 urological cases. Other previously published urological case series included only 18 (7), 53 (8) and 58 (9) patients, making our case series quite relevant in the literature.

The most frequently performed procedure in this case series was RARP (183 procedures), accounting for 60.6% of the total. The mean docking time for this procedure was 12.99 minutes, and for console time, it was 196.7 minutes. These results were similar to the case series by De Maria et al. (7) and Polom et al. (9) but lower when compared to that of Soumpasis et al. (6). The nephroureterectomy procedure required the shortest docking time (8.7 minutes, SD 2.8). This result was possibly due to the similarity of the arrangement of the Versius® trocars with the pure laparoscopic approach, which is widely known by surgeons, and to the greater ease of performance of this procedure due to the greater spacing between the trocars in nephroureterectomy compared to nephrectomy, easily adapting to any patient biotype.

The Versius®, being modular, allows for using three or four BSUs during the surgical procedure. Both possibilities of BSUs with different trocar arrangements have been validated in preclinical studies, including for RARP and nephrectomies, specifically (10, 11). In this case series, 270 (89.4%) procedures used four BSUs, which is of great value since the fourth arm is one of the advantages of robotic over laparoscopic surgery. The Versius® adapts to the patient and the team, and our results show no difference between docking and console time for obese (p=0.507) and non-obese (p=0.733) patients. Due to be modular, a fast undocking of the BSUS in case of emergency was observed in the only conversion to open surgery described. We considered a great advantage of robotic modular platforms.

Regarding the arrangement of the BSUs, the most prevalent was three on the left and one on the right of the patient (25.85%) when in pelvic procedures. This result demonstrates that the teams mostly preferred the third robotic arm in the surgeon's left hand and the bedside assistant on the patient's right side, as in the first description of docking by Rocco B et al. (12).

It is important to point out that properly evaluating robotic surgical techniques is complicated by robots being a "disruptive" technology, changing every aspect of the work environment. Indeed, clinical evaluation of safety and efficacy is only one of many dimensions that must be included in a comprehensive assessment of their overall value (13). In broad alignment with the Idea, Development, Exploration, Assessment, Long-term study- Devices framework, evidence detailing the development of the device has been reported in previous studies validating the usability and operational safety of Versius® (3, 5, 6). Preclinical studies have reported the successful use of the device in multispecialty including urological procedures in cadaver and porcine specimens (10, 11). Subsequently, live human clinical studies have evaluated the safety and effectiveness of the device for use in various multispecialty procedures (6–9, 12, 14). We report the largest analysis of urologic cases across a range of surgical indications. Data were reported as a measure of device and surgeon performance and the unprecedented real-world data presented here provide evidence of the safe implementation of a next-generation surgical robot into clinical practice, especially in Brazil.

Previous studies have identified a correlation between surgeons’ prior experience and their performance in training on robotic surgical systems (9, 11, 14–17). Butterworth et al. (15) demonstrated that surgeons with extensive experience in robotic surgery performed better in the Versius® training program than surgeons without experience, especially in the depth perception and robotic control domains. On the other hand, some metrics provided by studies using the Versius® simulator suggest that surgeons with prior experience in robotic surgery may take longer to adapt to certain specific Versius® techniques than those without any robotics experience. This result is mainly due to the features of the Versius® that differ from other platforms, such as the hand controller grip and the lack of foot pedals for electrocautery control (6, 8, 15).

Regarding the learning curve, this study demonstrated a decrease in docking time as the curve developed for 6 of the 10 (60%) surgeons who performed 10 or more procedures, while console time decreased for 4 of these same 10 (40%) surgeons. Notably, the type of surgery and the surgeon's previous experience were not considered, which may elucidate why docking time showed a greater reduction with statistical significance than console time. Butterworth et al. (15) suggested that adapting Versius® training to the surgeons’ previous experience may facilitate and reduce the learning curve since previous routine with other platforms may have contributed to a longer path in adaptation and learning curve with this platform. On the other hand, the similarity of some aspects, such as the arrangement of the trocars and the grip of the hand controllers, facilitate surgeons with experience in laparoscopy.

Concerning professional experience, 180 (59.6%) procedures were performed by surgeons who reported having experience in laparoscopy and/or robotic surgery for at least 5 years before using Versius®. Although only 7 of the 19 surgeons (36.7%) had performed more than 10 surgeries, the vast majority of the total surgeries (238 surgeries, 78.8%) were performed by these surgeons. In this sense, surgeons experienced in laparoscopy and/or robotics adapt more easily to this platform, as described by other authors (3, 15). Furthermore, this result corroborates the validation of our results regarding the learning curve for using the Versius® platform.

Our study has some limitations: a) the surgeries included does not represent 100% of surgeries performed with Versius® in the period of this study because a small number of surgeons did not include their surgeries in the database, b) our study is not a comparable trial head-to-head with Da Vinci® platform, c) difficulty of comparison to the literature because the few and small urological series published with Versius®.

Versius® exhibited efficacy and safety with comparable results to other robotic platforms in a systematic review that included publications on preclinical use in different series with human cadavers and swine models (18) and also included several clinical case series in humans (6–9, 19). Notably, our results concerning complication rate, length of hospital stay, reoperation rate and hospital re-admission are consistent with the literature, specially according to new platforms (20–24). Thus, given the scarcity of publications of urological case series with the Versius® platform, we can conclude that these perioperative results are similar and comparable to those already published with other multiport platforms (2). In addition to being the largest global urological case series with Versius®, it is worth mentioning that this study includes most of the urological procedures that can be performed with robotic technology with outcomes similar to those reported in the literature.

## CONCLUSIONS

This study reports the largest Brazilian and global series of urological surgeries performed with the Versius® robotic platform. The perioperative outcomes reported here, including a wide range of uro-oncological surgeries, demonstrate this platform's safety and efficacy and provide real-world evidence of the learning curve and performance of the surgeons according to their prior expertise in laparoscopic and/or robotic surgery.

## ABBREVIATIONS

BMI: = Body Mass Index
RALP: = Robotic Assisted Laparoscopic Prostatectomy
RASP: = Robotic Assisted Simple Prostatectomy
MAS: = Minimal Access Surgery
BSU: = Bedside Units
